# Characteristics of drowning victims in a surf environment: a 6-year retrospective study in southwestern France

**DOI:** 10.1186/s40621-019-0195-x

**Published:** 2019-05-13

**Authors:** Éric Tellier, Bruno Simonnet, Cédric Gil-Jardiné, Bruno Castelle, Marion Bailhache, Louis-Rachid Salmi

**Affiliations:** 1 INSERM, ISPED, Bordeaux, France; 2 Univ. Bordeaux, ISPED, Bordeaux, France; 3 CHU Bordeaux, Pôle Urgences Adultes-SAMU-SMUR, place Amélie Raba-Léon, 33076 Bordeaux, CEDEX France; 4 CNRS, UMR EPOC, Univ. Bordeaux, Bordeaux, France; 5 CHU de Bordeaux, Pôle de Pédiatrie, 33076 Bordeaux, France; 6 CHU de Bordeaux, Pôle de santé publique, Service d’information médicale, 33076 Bordeaux, France

**Keywords:** Drowning, Epidemiology, Natural hazard

## Abstract

**Background:**

Drowning is the third cause of non-intentional injury death worldwide. Beaches of Gironde, in southwestern France, are exposed to strong environmental conditions, leading to rip currents and shore breaks. Bathing season usually lasts from April to October and is supervised from June till mid-September. The objective of this study was to study the characteristics of drowning victims along Gironde surf beaches and to identify peculiarities compared to national figures.

**Methods:**

All calls originating from beaches to the emergency call center of Gironde from 2011 to 2016 were analyzed. Patient data, filled by a physician based on information given by pre-hospital care team (lifeguards, paramedics or emergency physicians), were extracted from the emergency call center database. We used Szpilman classification (0 = rescue to 6 = cardiac arrest) to assess severity. Rescues are patients without respiratory impairment who needed lifeguards or helicopter intervention. We compared our findings with national studies carried every three years (2012 and 2015).

**Results:**

We analyzed 5680 calls from beaches and included 4398, 576 of which were rescued from the water, including 352 without respiratory impairment (stage 0). Among drownings, 155 had cough only (stage 1), 26 pulmonary rales (stage 2), 9 pulmonary edema (stage 3) and 1 had pulmonary edema with hypotension (stage 4). Five rescued people were in respiratory arrest and 28 were in cardiac arrest. 77.5% were bathers, others were mainly surfers or body-boarders. Drowning victims median age was 24 (quartiles: 17–40), and sex-ratio was 1.44 Male/Female. Men were significantly older than women (34 vs. 26 years old), and severity from stage 1 to 4 was positively associated with age. Compared to national data, Gironde drownings had a higher proportion of 15–44 year-old victims, and the case-fatality was lower in Gironde (11.5%) than at the national level (27.4%, *p* < 0.001).

**Conclusion:**

Along Gironde coast, drowning is rarely severe, concerns mostly young men; the age distribution could explain the different case-fatality. Further study is needed to identify environmental predictors of drowning.

## Background

Drowning is defined in 2005 by the World Health Organization (WHO) as “the process of experiencing respiratory impairment from submersion/immersion in liquid” (van et al., 2005). It is the third leading cause of non-intentional injury death worldwide, representing in 2017 294,000 people according to the Global Burden of Disease data (Dicker et al., 2018).

**Fig. 1:**
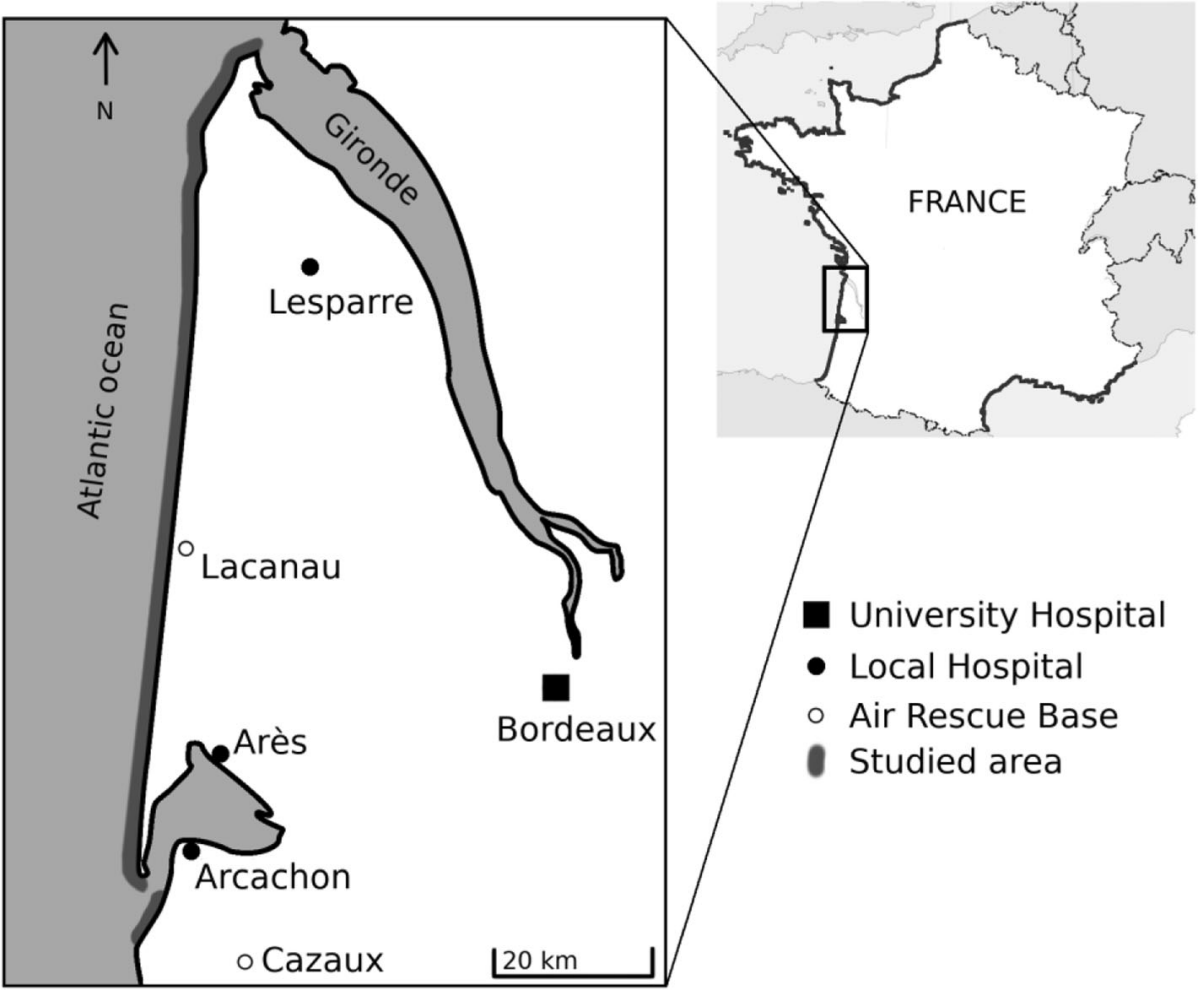
Map of Gironde, south western France, with description of the studied area and nearby hospitals

In France, every three years, a national study is conducted by the French national institute of public health (Santé Publique France), registering all cases of drowning leading to hospitalization or death, from June, 1st till September, 30th. During 2015, this study reported 1266 drownings (fatal and non-fatal) in France, including 637 (50.3%) on coasts (Lasbeur et al., 2016); data were similar in the 2012 report (Lasbeur & Thélot, 2013). Studied coasts are highlighted in the overall map of Fig. 1 (Corsica not shown). On the beaches of Gironde, an area in Southwestern France, magnified in Figs. 1, 38 drownings resulting in 7 deaths were reported in the 2015 study.

Drowning is associated with both environmental and individual factors, and occurs quickly. Although few interventions have been evaluated, prevention is considered key to improve beachgoers behavior in specific environmental conditions and hopefully reduce drownings (Bierens, 2006; Hatfield et al., 2012; Sherker et al., 2010; Szpilman et al., 2012). Once drowning occurs, a chain of survival implies a fast response with paramedics and a medical team if needed (Dyson et al., 2013; Salomez & Vincent, 2004; Szpilman et al., 2014). Indeed, watched areas needed less medical attention and cardiopulmonary resuscitation for victims of drowning (Szpilman, 1997; Venema et al., 2010).

The sandy coast of Gironde is exposed to high-energy waves. This leads to a ridge and runnel system inducing rip currents (Castelle et al., 2007). These conditions can be encountered in other parts of the world, and it is known that most beach-related drowning are caused by rips (Castelle et al., 2018; Gensini & Ashley, 2010; Morgan et al., 2008).

The Gironde coast comprises 126 km of sandy surf beaches. Because it is a major touristic destination in southwestern France, beach safety is an important concern. During the high season, from mid-June to mid-September, major beaches are watched by lifeguards from 11:00 AM to 7:00 PM. Some of the busiest beaches are watched from the beginning of June, but bathing season can begin in April and last till the end of October when warm weather conditions prevail. Drowning prevention is done essentially within guarded areas, through signage at the beach entrances and distribution of leaflets describing rip-current and shore-break hazards which are the primary surf zone hazards along this stretch of coast. During the high season, two helicopters are available for sea rescue.

Describing the drowning problem at a local and regional level is an essential step to develop effective prevention countermeasures (van et al., 2005; Idris et al., 2003; World Health Organization, 2014). There is a need of documenting history and description of victims in that particular environment. Moreover, as drowning occurs quickly, people rescued by lifeguards need to be taken into account, even if the absence of severe drowning stage did not lead to hospitalization.

The objective of our study was to characterize drowning victims in the oceanic coast of Gironde and to identify peculiarities compared to national figures.

## Methods

### Data source and inclusion criteria

During summer, lifeguard interventions for rescue from water is followed by a call to the Medical Emergency Call Center. This center also receives direct calls from beachgoers. This retrospective study was based on data from emergency calls coming from Gironde beaches from January 1st, 2011 to December 31st, 2016. As we were interested in drownings occurring in strictly surf environment, calls from the beaches inside the Arcachon lagoon (Fig. 1) were excluded. Collecting every call from beaches, even those unrelated to drowning, is also interesting as a previous, unpublished, work carried out in 2003 in Gironde reported a strong correlation during summer between beach attendance and number of calls for any reasons coming from beaches (Murcott, 2003).

All medical emergency calls from beachgoers or lifeguards come to a Medical Emergency Call Center (SAMU, Service d’Aide Médicale d’Urgence). For each call, a physician fills the database with information given by callers, and paramedics and pre-hospital care teams when involved. We excluded calls without victim, trainings and duplicates. Contents of all calls were reviewed by the first author, a junior epidemiologist with training in emergency medicine. We then classified all calls in two groups, drowning cases and other calls.

### Variables and data collection

We collected location, date and time of the call, gender and age of the victim for all medical emergency calls from beaches. For drownings, we collected the medical history of the victim, activity (bathing, surf, bodyboard, other), initial status given by lifeguards report, and discharge status (left on site, transferred to emergency department or intensive care unit, death on site, death at hospital). The severity of drowning was rated, from the first clinical description reported, with the Szpilman classification (Table 1). Stage zero, or rescue, characterizes people who need to be rescued from water, but without cough. Stage 1 is defined by the presence of cough, stage 2 victims present pulmonary rale in some fields; stage 3 have pulmonary edema and stage 4 present pulmonary edema with hypotension. Stage 5 are patient with respiratory arrest without cardiac arrest and stage 6 is cardiac arrest. Stages from 2 to 6 need to be hospitalized while stage 1 can be left under surveillance of lifeguards. When available, we recorded resources such as helicopter or jet-ski used to rescue the victim. Finally, location of event (in or out bathing area, beach location) was recorded.

**Table 1:** Short description of drowning stages, according to Szpilman classification (Szpilman et al., 1997 and 2012)

Stage	Symptomatology
0 (rescue)	None
1	Cough, normal pulmonary auscultation
2	Cough, rales in some pulmonary fields
3	Acute pulmonary edema with normal blood pressure
4	Acute pulmonary edema, hypotension or shock
5	Respiratory arrest and/or coma
6	Cardiac arrest

We assumed that stage-0 and -1 victims without hospital transfer had a favorable outcome. Outcomes of the most severe victims (stage 2 and over) were collected from the files of Intensive Care Units were patients were admitted. National data, collected every three years, were extracted reports of the national institute of public health, Santé Publique France, carried in 2012 and 2015 (Lasbeur et al., 2016; Lasbeur & Thélot, 2013). Those data are gathered using hospital (emergency and pre-hospital care services) and emergency call center reporting of drownings leading to hospitalization (including emergency evaluation) or death.

### Statistical analysis

We first compared calls for rescue and drowning with other calls to characterize the studied population. We then compared our findings on drownings to national data. To describe drowning victims, qualitative variables were reported as percentages and numbers. Quantitative variables were reported as medians and inter-quartile range. Fisher and permutation tests were used to assess differences among groups for qualitative and quantitative variables respectively. As spatial distribution of accesses to the beaches is discontinuous and mostly depends on municipal boundaries, continuous spatial data analysis was not possible, and we divided the coast into 9 areas (from A to I in Fig. 1) according to those boundaries.

We calculated univariate odds ratios of drowning occurrence among calls between low- and high-seasons, between weekdays and weekend and between locations. Data analysis was done with the R software and coin package (R Core Team, 2017; Harrell, 2014; Harrell, 2015; Hothorn et al., 2008).

## Results

### Emergency calls and their characteristics

**Fig. 2:**
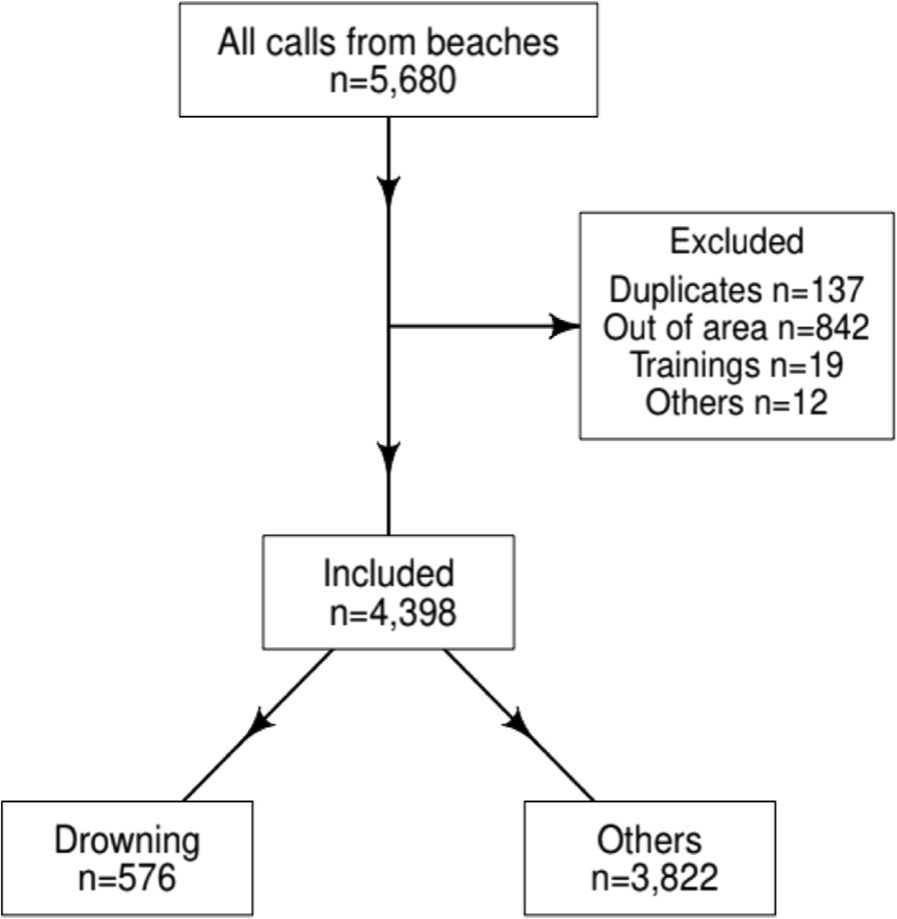
Flow chart of call data from the surf beaches to the emergency medical service center of Gironde, France, during years 2011–2016

We screened 5680 call files, of which 1282 were excluded (Fig. 2). For calls secondary to rescue from water, age was missing in 170 files (29.5%), mostly for stages 0 (*n* = 164) and 1 (*n* = 6) patients. Missing data concerned helicopter interventions for air-lifting operations where, usually, only the number of involved patients, their severity, and sometimes sex are filled. Median age was 24 years (mean 30.9, quartiles: 17–45) among people rescued from water and 23 years among other calls (mean 28.4, quartiles: 15–40). Compared to other beach-related calls, the sex-ratio was not different (*p* = 0.88). Males rescued from water were older than for other beach-related injuries (*p* < 0.001), but female victims were similar in age (*p* = 0.06).

**Fig. 3:**
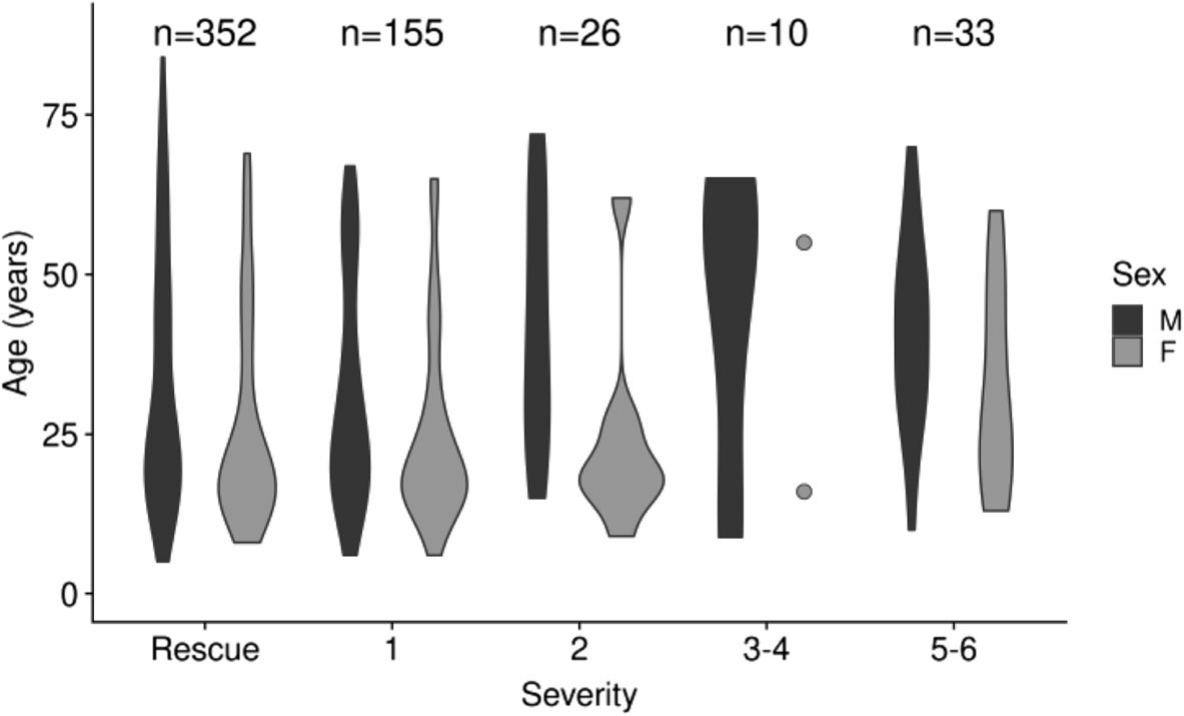
Age of drowning victims according to sex and severity, Gironde surf beaches, 2011–2016. Width is proportional to number of individuals in group at a given age. Only 2 females were reported for stages 3 or 4

Sex was available for 427 cases (74.1%) and most victims were stages 0 (61.1%) and 1 (26.9%) (Table 2). Other calls (*n* = 3822) were mostly secondary to medical cause, beach-related trauma, but there were specific surf injuries such as shore-break related injuries (17.7%) and envenomation (5%) (Table 3). Among calls secondary to rescue from water, most patients were males (M/F = 1.44) and the severity was not different in males and females (Fig. 3, *p* = 0.14). Males were significantly older than females (mean 34 vs. 26 years, *p* < 0.001). Age varied across stages, independently of sex (*p* = 0.001) (Fig. 3). Low- and high-season drownings were not different in age distribution, but a higher proportion of males were victims during low season (Odds Ratio (OR) 2.42 95% confidence interval (CI) [1.39–4.31], *p* < 0.001).

**Table 2:** Population characteristics of calls secondary to rescue from water (*n* = 576) along Gironde surf beaches, 2011 to 2016. Severity was assessed according to the Szpilman classification (Szpilman et al., 2012)

Variable	N	%
Sex		
Female	175	30.4
Male	252	43.6
Missing	149	26.0
Severity		
0 (Rescue)	352	61.1
1	155	26.9
2	26	4.5
3	9	1.6
4	1	0.02
5	5	0.9
6	28	4.9
Outcome		
Left on site	363	63.0
Death	20	
Evacuated		
University hospital	20	3.5
Death	4	
Local hospital	163	28.3
Other hospital	2	
Missing	8	
Activity		
Bathing	444	77.5
Body board	11	1.9
Surf	24	4.2
Other	3	0.5
Missing	91	15.9

**Table 3:** Population characteristics of emergency calls excluding victims of drowning from Gironde surf beaches, 2011 to 2016 (*n* = 3822)

Variable	N	%
Sex		
Female	2133	55.8
Male	1673	43.8
Missing	16	0.4
Shore-break related injuries	675	17.7
Shoulder	177	
Neck	177	
Back	96	
Knee	96	
Head	42	
other	87	
Envenomation	191	5.0
Weevers	117	
Jellies	25	
*Physalia physalis*	34	
Missing	15	
Dislocation	235	6.1
Wounds	525	13.7
Other trauma	1059	27.7
Miscellaneous Other Medical	959	25.1
Seizure	35	0.9
Missing	146	3.8
Activity		
Bathing	282	9.0
Body board	88	2.8
Surf	769	24.4
Other seashore activity	39	1.2
Activity on the beach	907	28.8
Missing	1062	33.7

A medical history was explicitly noted for 49 cases (8.5%), reporting mainly cardio-vascular diseases (*n* = 17), asthma (*n* = 13), and psychiatric disorders (*n* = 5); one patient had a history of epilepsy. The absence of medical history was documented in 112 cases (19%). Therefore, 415 patients (72.0%) were without reporting medical history. Alcohol consumption was notified in 2 cases. No drowning occurred within bathing area during the study period.

**Fig. 4:**
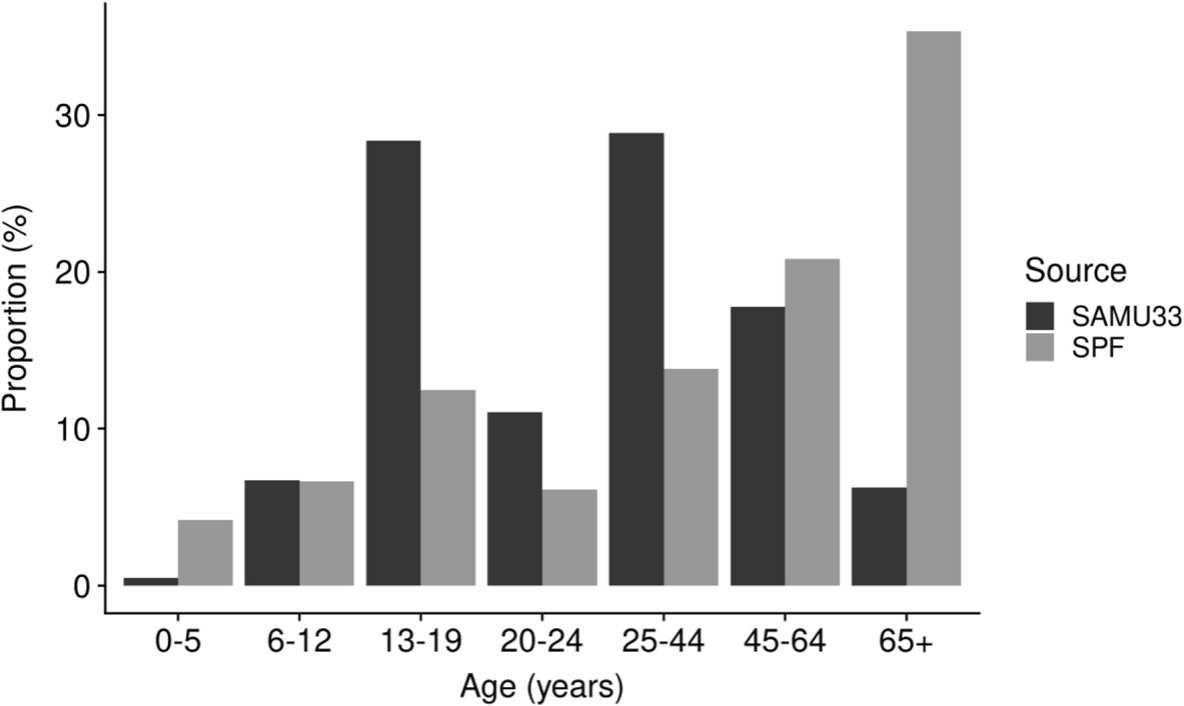
Comparison of age of drowning victims who needed a hospitalization or died, from the database of Gironde Emergency Call Center, years 2011–2016 (SAMU33, *n* = 188) and along all coasts of France (SPF, data from Santé Publique France, French national institute of public health, reports of 2012 and 2015, *n* = 1165)

Compared to national data, Gironde drownings had a higher proportion of 15–44 year-old victims and a lower proportion of people above 45 and below 5 (Fig. 4). The case-fatality was lower in Gironde than at the national level (11.5% vs 27.4%, p < 0.001).

### Drowning timing and location

With 118 rescues from water and drowning events reported at the emergency call center with 556 calls for other beach-related events, there were significantly more rescues leading to a call in 2014 than in other years (Table 4). Over six years, the number of drownings varied depending on whether the day was a weekday, a weekend or a holiday. During the low-season, the probability that a call concerned a drowning case was increased on weekdays (OR 3.11; 95% CI 2.07–4.62) even though 7 deaths occurred on weekends and 2 during weekdays in low season. Adjusted to the other calls, the low season was riskier than the high season (OR 1.98; 95% CI 1.54–2.52).

**Table 4:** Characteristics of drownings compared with other calls from beaches of Gironde, by type of day, year, and location with odds ratio (OR) and 95% confidence intervals

Variable		Other calls	Drownings	OR	95% CI
Type of day					
	Holiday	52	11	1.41	0.66–2.75
	Week-end	205	44	1.46	1.02–2.06
	Weekdays	87	40	3.20	2.12–4.76
	High season	3.478	481	0.50	0.39–0.65
Year					
	2011	670	88	0.85	0.66–1.08
	2012	708	116	1.11	0.88–1.39
	2013	617	70	0.72	0.54–0.94
	2014	556	118	1.51	1.20–1.90
	2015	664	88	0.86	0.67–1.10
	2016	607	96	1.06	0.83–1.35
Location					
	A	292	40	0.90	0.62–1.28
	B	105	20	1.27	0.74–2.09
	C	337	46	0.90	0.64–1.24
	D	161	26	1.07	0.67–1.65
	E	293	58	1.35	0.98–1.82
	F	1217	160	0.82	0.67–1.00
	G	199	51	1.77	1.26–2.45
	H	760	152	1.44	1.17–1.77
	I	458	23	0.31	0.19–0.47
Total		3.822	576		

The proportion of drowning among emergency calls was lower in area I than in other locations (OR 0.31; 95% CI 0.19–0.47). Areas G and H had a significant higher incidence than other locations (Table 3).

## Discussion

To our knowledge, this is the first study that characterizes precisely the burden of drowning in a high-risk area, taking into account both rescues and off-season drownings. The latter do not usually appear in lifeguard registries as they work only during high-season (mid-June to the end of August for most stations). This study is the first step to prevention, analyzing existing data in a specific context (World Health Organization, 2017) to define and orient targeted prevention strategies, according to WHO and CDC recommendations (World Health Organization, 2017; Doll et al., 2007). Such data collection could be done in other area for comparison.

Our study has some limits related to the retrospective use of existing medical files. Although records of the Emergency Call Center are useful to identify drownings needing medical advice or helicopter interventions, they miss rescues by lifeguards that did not lead to a call for medical advice. Local regulation requires lifeguards to report all victims, but it is known that there are missed cases as some local annual unpublished reports, show a higher number of rescues than our study. Therefore, one has to be careful when making external comparison because the number of calls is only an indirect estimation of the denominator. Further, Medical Emergency Call Center files were not always documenting medical history and circumstances of drowning. They were still filled well enough to assess initial severity of drowning.

Data collection of beach-related injuries, including drowning, is challenging (Williamson, 2006). We tried to access to local data, but they are incomplete or very scarce, reporting only total numbers of rescue for a given day for example. Thus, we do not have any sources on demographics of bathers. Following this study, the identified need for a better documentation led to the development of a specific questionnaire, which was distributed to Gironde lifeguards at the start of the 2018 season.

Men were less represented than in most epidemiological studies (Gensini & Ashley, 2010; Claesson et al., 2014; Claesson et al., 2012; Clemens et al., 2016; Harada et al., 2011; Quan et al., 2014), but the male/female ratio is consistent with other oceanic studies (Szpilman et al., 2014; Doll et al., 2007; Williamson, 2006; Claesson et al., 2012). In our study, drowning victims were mostly adolescents and young adults, with very few children. This low proportion of children compared to other drowning epidemiological studies might be due to a lower exposure: they may be more likely under supervision of a parent, or less exposed to surf beaches as lakes are common in the area. Indeed, most studies do not focus on surf environment. Nevertheless, this age distribution is not surprising given the wave conditions, with frequent shore breaks; the age distribution is similar to findings in Hawaii (Williamson, 2006) and Canada (Doll et al., 2007). Compared to other calls, people rescued from water were older, especially among males. This could be explained by a riskier behavior among adult males. In Australia, similar findings have been reported regarding the propensity to swim outside flagged area (Sherker et al., 2010), and death rate among surf beach swimmer population (Morgan et al., 2008).

The comparison to national data shows that the surf environment has a particular population and suggests a need to identify geographical disparities along the coastline. Compared to other coastal areas contributing to national data, Gironde is known to be a place with many rip current occurrences and strong shore-break waves. Those hazards can be found in all other coasts of France, but with much less frequency and intensity. Water temperature is generally lower than along Mediterranean coasts, and higher than in Brittany and northern coasts. Air temperatures are similar to Mediterranean coasts. Unfortunately, we do not have similar studies to compare demographics of victims in other places, but the demographic distribution could explain the difference of death rates between national and regional data.

We could not differentiate locals from tourists, which could imply a different prevention strategy, but previous studies along southwestern coast of France reported a majority of non-locals but few foreigners (Castelle et al., 2018). Nevertheless, other studies suggest a higher risk of drowning among non-residents (Williamson, 2006). Severity of drowning was correlated with age in stages 1 to 4, but not for the severe stages 5 and 6, which is consistent with other studies (Dyson et al., 2013; Quan et al., 2014; Morgan & Ozanne-Smith, 2013; Suominen et al., 2002). Our data did not allow studying predictive factors of outcome.

The total number of beachgoers and bathers is unknown, thus precluding estimating the actual exposure. To compensate for this lack of denominator data, Gensini et al. suggested using the number of deaths by 100-km segments of coastline (Gensini & Ashley, 2010). Dividing the US coastline in 100-km segments to examine rip-current fatalities, they found the highest risk in eastern Florida, with 46 fatalities in 14 years for 100 km. This gives a normalized yearly rate of 3.3 deaths/100 km. In our study, the normalized death year ratio in Gironde would be 3.2 deaths/100 km, showing a risk equivalent to the highest observed in the U.S.A. Further studies are needed to measure the number of beachgoers and bathers and therefore provide a better denominator to estimate the incidence of drowning.

Most victims were rescues and stage 1, with few medical needs, although they needed lifeguards and, sometimes, helicopter interventions. As drowning occurs quickly, those victims need the same focus of prevention than the others. Missing cases would very likely concern rescues or stage 1. Our study is a first step to collect data from this population. In the absence of adequate exposure estimations, a prevention strategy targeting the whole population and not only the most severe cases, might be more feasible and effective.

Several results can help us orient a possible prevention strategy. Most drownings occurred during high season but, adjusted to the other calls, low seasons and weekdays showed more calls for drowning or people rescued from water. This could be due to lifeguards prevention which is a major activity (Koon et al., 2018), or a bias concerning the other calls. On the other hand, hot weather is known to be associated with drowning (Dyson et al., 2013). Therefore, prevention messages should be more reinforced in the absence of lifeguards, and lifeguard presence could be adapted.

Alcohol appeared in very few cases (0.3%), compared to other studies (Szpilman, 1997). This could be a reporting bias, but also explained by the fact that most beaches are in remote areas and are unlikely to be frequented after sunset.

The number of calls is an indirect measure of the denominator, but further work is needed to ensure the comparisons between years and seasons. Year 2014, with a higher proportion of calls concerning rescues from water, is an outlier. To our knowledge, there were no difference in lifeguard headcount, nor in safety campaigns, and weather data from MétéoFrance showed normal summer temperatures, except a cold end of August. This difference could be explained by winter storms and spring weather that can fundamentally change the shape of the beach and therefore the occurrence of rip currents (Castelle et al., 2016).

The spatial distribution of drownings along the Gironde surf coast also shows some interesting significant disparities. The higher risk of areas G and H could be due to the proximity with the metropolitan area of Bordeaux, with a higher exposure. Another explanation is the difference of watching conditions between municipalities, as there can be twice as many lifeguards per kilometers of beach in a given area than in another. A missing parameter is the proportion of rescues within the bathing area, but our data show that all deaths occurred outside these watched areas.

Geographic disparities are a supplementary strong argument for rip current hazards: area I, less exposed to waves, had the lowest incidence of rescue from water among calls. This could not be explained by difference in beach access (it is near a city) neither by the amount of lifeguards (comparable to area H). As rip currents are driven by wave and tide conditions (Castelle et al., 2016; Bruneau & Castelle, 2009), a future study is needed to explore the link between rescues and environmental parameters.

## Conclusion

This observational study in an area with specific hazards identified the demographics of drowning victims and led to the development of a specific questionnaire to improve reporting. This study revealed notable differences with national data and suggests that drownings along coastline must be studied at a local scale. The studied area is at high-risk of rip-related drowning and there is a need to identify days at risk using meteorological, wave and tide parameters. Such a study could lead to a prevention tool for lifeguards and emergency managers.

## Abbreviations

CDC: Center for Disease Control
CI: Confidence Interval
OR: Odds Ratio
SAMU: Service d’Aide Médicale Urgente (emergency medical service)
WHO: World Health Organization
